# Spatial distribution of maternal factors in pig mature oocytes

**DOI:** 10.1080/10495398.2024.2394692

**Published:** 2024-08-26

**Authors:** Fuquan Zhu, Meng Yang, Dayu Wang, Yuan Jiang, Chao Jia, Yanfeng Fu, Aochen Yu, Huijun Liu, Meixia Wang, Tingzhang Wang, Honglin Liu, Juan Li

**Affiliations:** a College of Animal Science and Technology, Nanjing Agricultural University, Nanjing, China; b Institute of Animal Science, Jiangsu Academy of Agricultural Sciences, Nanjing, China; c Key Laboratory of Microbial Technology and Bioinformatics of Zhejiang Province, Zhejiang Institute of Microbiology, Hangzhou, Zhejiang Province, China

**Keywords:** Porcine, transcriptomics, proteomics, maternal factors, oriented bisection

## Abstract

It is known that asymmetrical maternal transcripts play an important role in the cell fate of the early embryo, but few studies are available in mammal oocytes especially in pig. To investigate the spatial factors in pig oocytes, the oriented bisection was established for collecting karyoplasts (NSOs) and cytoplasts (SSOs) with more than 95% efficiency. Subsequently, RNA-Seq and LC-MS/MS analysis were performed on NSOs and SSOs. Although no differentially expressed genes (DEGs) could be detected between NSOs and SSOs, 89 of the differentially expressed proteins (DEPs) were detected, that 58 proteins higher expressed but 31 proteins lower expressed in NSOs compared with SSOs. These DEPs mainly participated in the ‘cell cycle’ and ‘ribosome’ pathway, while the up-regulated DEPs were mainly GO in ‘spindle’ and ‘positive regulation of translation’, and the down-regulated DEPs were in ‘cytosolic small ribosomal subunit’ and ‘mRNA binding’. The up-regulated DEP SIRT5 which are related to the regulation of gene expression, epigenetic were further detected and revealed. A spatial asymmetry of maternal factors at the protein level was firstly detected in pig mature oocytes.

## Introduction

Maternal factors are special substances synthesized and stored in oocytes during oogenesis, which will exert their main role during fertilization and preimplantation development.^1^ The fully differentiated meiotic metaphase II (MII) oocyte, prior to fertilization, has often been characterized as ‘polarized’, based on an apparent morphological asymmetry where in the metaphase chromosomes are localized.^2^^,^^3^ Accumulating evidence suggested that the uneven distribution of RNAs and proteins are also noticed in mammalian oocytes,^4–6^ such as an RNA population contained in the nucleus that most likely contributes to the translation in the vicinity of chromosomes after nuclear envelope break down.^7^ The enriched mRNAs in the spindle area^8^ might function for the formation, maintenance, function, and other nearby processes of the spindle.^9^ In addition, the asymmetric distribution of some certain mRNAs and proteins related to pluripotency and lineage commitment have been confirmed in the oocytes of sheep and bovine by orientated bisection.^4^^,^^10^ Asymmetric localization of maternal factors (including mRNA and protein) in oocytes, which are not evenly distributed but regionally organized in most invertebrates and amphibians, is a fundamental polarity determinant for cell fate in developing embryos.^11^^,^^12^ Therefore, exploring the distribution of maternal factors is crucial for understanding key events in oocyte meiosis, fertilization, and early mammalian development.

As the main cytoplasmic recipient for somatic cell nuclear transfer (SCNT), the cytoplast is one of the crucial factors affecting the efficiency of SCNT embryos.^13–15^ To get enough cytoplasts by enucleation, the nucleus in the matured oocyte was aspirated out by the micromanipulation in traditional cloning or was cut off by the oriented bisection in handmade cloning (HMC).^16^^,^^17^ When the reconstructed embryo was produced by the fusion of the cytoplast and donor cell, the M-phase promoting factor (MPF) present in the cytoplasm triggered the rapid nuclear envelope rupture of the donor nucleus, forming condensed metaphase-like chromosomes.^18^ Subsequently, the reprogramming factors in the cytoplasm reversed cell fate into the pattern of a totipotent zygote through the metabolic switch, chromatin remodeling, and global epigenetic transformations.^19^^,^^20^ To date, various species have been successfully cloned by SCNT, however, both the efficiency of SCNT and the developmental competence of cloned embryos were relatively low, and even abnormal offspring born.^21^^,^^22^ During enucleation of SCNT, some specific mRNAs and proteins might be lost when the karyoplasm part was removed, such as maternal factors of *HDAC3*, *TRIM28* and some regulators of the spindle,^23^^,^^24^ which might lead to the incomplete reprogramming or epigenetic abnormalities of SCNT embryos.^25–27^ Therefore, it is crucial to have more knowledge about the distribution of maternal factors including mRNAs and proteins in the matured oocytes.

The present study was designed to investigate the distribution of maternal factors in pig matured oocytes. Firstly, in order to collect the half oocytes of karyoplast (NSOs) and another half oocytes of cytoplast (SSOs), the oriented bisection of oocytes was performed according to the position of the first polar body (PB1) was established and modified from HMC.^28^^,^^29^ Subsequently, differences of mRNAs and proteins in NSOs and SSOs of pig MII oocytes were analyzed.

## Materials and methods

### Chemicals

All chemicals were purchased from Sigma-Aldrich Co, Inc. (St. Louis, MO), unless otherwise indicated.

### The oriented bisection of pig matured oocytes for collecting NSOs and SSOs

**Figure 1.**
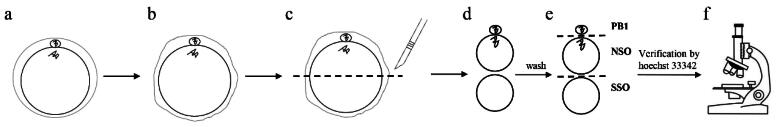
Collection and the quality control of NSOs and SSOs. (A) MII oocyte with PB1. (B) Partial digestion of ZP. (C) Oriented bisection was performed on the oocyte. The dotted line represented the position when the oriented bisection was performed. (D) One oocyte was separated into two semi-oocytes of karyoplast and cytoplast, which were collected as NSO and SSO (E), respectively. (F) Verification of the nucleus by the staining of hoechst 33342 to calculate the positive rate of NSO and SSO.

Ovaries obtained from slaughterhouses were transported to the laboratory in 0.9% NaCl at 37 °C. Cumulus cells-oocyte complexes (COCs) with at least three layers of compact cumulus and even cytoplasm were selected for in vitro culture (IVC) in HEPES-buffered Tissue Culture Medium 199 (TCM-199) supplemented with 10% (v/v) fetal bovine serum (FBS), 10% (v/v) porcine follicular fluid (PFF), 0.8 mM L-GlutaMAX, 0.242% (v/v) gentamicin, and 15 IU/mL HCG, 15 IU/mL PMSG, in four-well dishes (Nunc, Thermo Fisher) at 38.5 °C, 5% CO_2_ with saturated humidity. After the removal of cumulus cells (CCs) of COCs by using 1.0 mg/mL hyaluronidase, 3.3 mg/mL pronase was used to soften the zona pellucida (ZP) of mature oocytes containing PB1 and perform oriented bisection as shown in Fig. 1(A–C). The whole oriented bisection process did not exceed 1h. After washing twice in PBS to remove PB1 and ZP, the half part karyoplast with a nucleus was collected as NSO, another part cytoplast without a nucleus was selected as SSO (Fig. 1D,E). Subsequently, the collected NSOs or SSOs were checked under the microscope with UV light after being stained with 10 µg/mL Hoechst 33342 (Fig. 1F).

### RNA isolation and library construction

In each group, 130 NSOs or SSOs were collected in PBS, which were stored in liquid nitrogen and were sent to Kaitai Biological Co., Ltd. (Hangzhou, China) for RNA-Seq in two replicates (NSO-R-1, NSO-R-2 and SSO-R-1, SSO-R-2). Following the manufacturer’s recommendations, RNA was isolated and amplified using SMART-Seq^™^ v4 Ultra^™^ Low Input RNA Kit, and sequencing libraries were generated using the NEBNext^®^ Ultra^™^ DNA Library Prep Kit for Illumina^®^ (NEB, Ipswich, MA, USA). Subsequently, the library quality was assessed on the Agilent 2100 Bioanalyzer system and sequenced using Illumina HiSeqTM 4000.

### Data processing

The clean data (clean reads) were obtained from the raw data (raw reads) of fastq format, by removing the low-quality reads, reads containing adapters, and reads containing ploy-N from raw data, processed through in-house perl-cripts. With the download of the pig reference genome (Sscrofa11.1) and gene model annotation files from Tophat (http://ccb.jhu.edu/software/tophat/index.shtml), a comparative analysis was performed on the clean reads and the reference genome.

### Bioinformatics analysis of gene expression

The read numbers mapped to each gene were counted using HTSeq (v0.6.1), as well as FPKM (fragments per kilobase of transcript sequence per million base pairs sequenced) of each gene were calculated based on gene length and read counts mapped to this gene by using RSEM (v1.2.12). Analysis of expression differences was conducted by using the DEG-seq function in Bioconductor, |log2Ratio| ≥ 1 and FDR-value ≤ 0.05 were considered differentially expressed genes (DEGs).

### Protein preparation

Approximately 520 NSOs or SSOs in each group were collected in PBS which were stored in liquid nitrogen for proteomic profiling in three replicates. Groups of NSO-P (NSO-P-1, NSO-P-2 and NSO-P-3) and SSO-P (SSO-P-1, SSO-P-2 and SSO-P-3) were lysed in lysis buffer containing 8 M urea and 1% protease inhibitor cocktail by ultrasonic waves, respectively. After centrifugation at 12,000 g at 4 °C for 10 min to remove the remaining debris, the protein concentration in the collected supernatant was determined with a BCA kit (Beyotime) according to the manufacturer’s instructions. Dithiothreitol (DTT) was added to the protein solution with the final concentration of 5 mM, and the reduction reaction was performed at 56 °C for 30 min, then iodoacetamide (IAA) was added to get a final concentration of 11 mM, with further incubation for 15 min at room temperature protected from light. To make sure the concentration was below 2 M with the addition of TEAB, a ratio of 1:50 (protease: protein, m/m) trypsin was used for enzymatic digestion overnight. Subsequently, another digestion of 4 h was continually carried out with a ratio of 1:100 (protease: protein, m/m) trypsin. Finally, the peptides were desalted by the C18 SPE column.

### LC-MS/MS quantitative proteomics

For liquid chromatography, the peptides prepared as described above were dissolved in mobile phase A (an aqueous solution of 0.1% formic acid and 2% acetonitrile) and were separated using the Nano Elute Ultra High-Performance Liquid Phase System. After separation by the ultra-performance liquid system with the gradient setting of 0–72 min, 7%–24% B (mobile phase B, a solution of 0.1% formic acid and 100% acetonitrile); 72–84 min, 24%–32% B; 84–87 min, 32%–80% B; 87–90 min, 80% B, while the flow rate was maintained at 400 nl/min, the peptide was injected into the capillary ion source for ionization, of which the voltage was set to 1.75 kV. The parent ions of the peptide and its secondary fragments were detected and analyzed by high-resolution TOF in timsTOF3 mass spectrometry, while the parallel cumulative serial fragmentation (PASEF) mode was used for data collection. After one primary mass spectrometry collection, the secondary spectrograms set to 100–1700 m/z with the charge number of parent ions in the range of 0–5 were collected in PASEF mode in 10 times. The dynamic exclusion time of tandem mass spectrometry was set at 30 s to avoid repeated scanning of parent ions.

### Database search and bioinformatics analysis

MS/MS data were processed using the MaxQuant search engine (v.1.6.15.0) by searching against Blast-Sus-scrofa-9823-PR-20210806.fasta (49,792 entries). Trypsin/P was designated as the lyase, allowing the two missing cleavages. The mass tolerance of the first and main searches for precursor ions was set at 20 ppm, while the mass tolerance for fragment ions was set at 0.02 Da. Carbamidomethyl on Cys was specified as a fixed modification, and acetylation of protein N-terminal and oxidation on Met were specified as variable modifications. For protein and peptide spectrum match identification, the false-discovery rate was adjusted to 1%. The proteins with a fold change (FC) >1.30 or < 0.76 and *p*-value < 0.05 were considered as the differentially expressed proteins (DEPs). The statistics of DEPs were analyzed by Perseus Version 1.6.15.0. The WolF Psort software was used to annotate the subcellular structure of proteins. Clusters of Orthologous Groups of protein/EuKaryotic Orthologous Groups (COG/KOG) were used for homologous prediction and functional classification. After the GO annotation of proteins with the UniProt-GOA database and the descriptions of annotated proteins, proteins were classified by Gene Ontology annotation based on three categories: biological process, cellular component and molecular function. KEGG online service tools KAAS, while the annotation results of KEGG pathways were mapped using KEGG mapper, DEPs were subjected to KEGG enrichment significance analysis (with the identified proteins as background) using Fisher’s exact test, as *p*-value < 0.05 was considered as the significant. All accession or sequence database of DEPs were searched against the STRING database (version 11.0) for protein-protein interactions (PPI) and the degree of connectivity was used as an evaluation index for screening key proteins. The results of which were visualized in Cystoscope software (version 3.8.2).

### Immunofluorescence staining (IF)

After brief washing in phosphate-buffered saline containing 1% BSA (PBS1), the matured MII oocytes with PB1 were fixed in 4% paraformaldehyde diluted in PBS1 for 30 min at room temperature (RT). After washing in PBS1 (5 min × 3 times), the oocytes were permeabilized with 0.5% Triton X-100 for 30 min at RT and blocked with PBS1 at RT for 2 h. For indirect immunofluorescence, the oocytes were incubated overnight at 4 °C with rabbit primary antibody against SIRT5 (Proteintech, 15122-1-AP) diluted 1:500 in PBS1. After washing (5 min × 3 times), the embryos were incubated at RT for 1 h with CoraLite488-conjugated Goat Anti-Rabbit IgG (H + L) secondary antibody (Proteintech, SA00013-2). After another three washes, embryos were treated with 5 mg/mL DAPI (Beyotime, C1002) for 15 min. As a negative control, the primary antibody was replaced with normal rabbit IgG. Subsequently, the oocytes were observed under a confocal laser scanning microscope (LEICA TCS SP8), while images of them were captured.

### Statistical analysis

All analyses were performed using Graph Pad Prism 8 (Graph Pad Software Inc., San Diego, CA, USA) with *t-test* analysis of variance. And data were presented as Mean ± Standard Error of the Mean (Mean ± SEM), while *p-*value < 0.05 indicated statistical significance.

## Results

### The high efficiency of oriented bisection for collecting NSOs and SSOs

**Figure 2.**
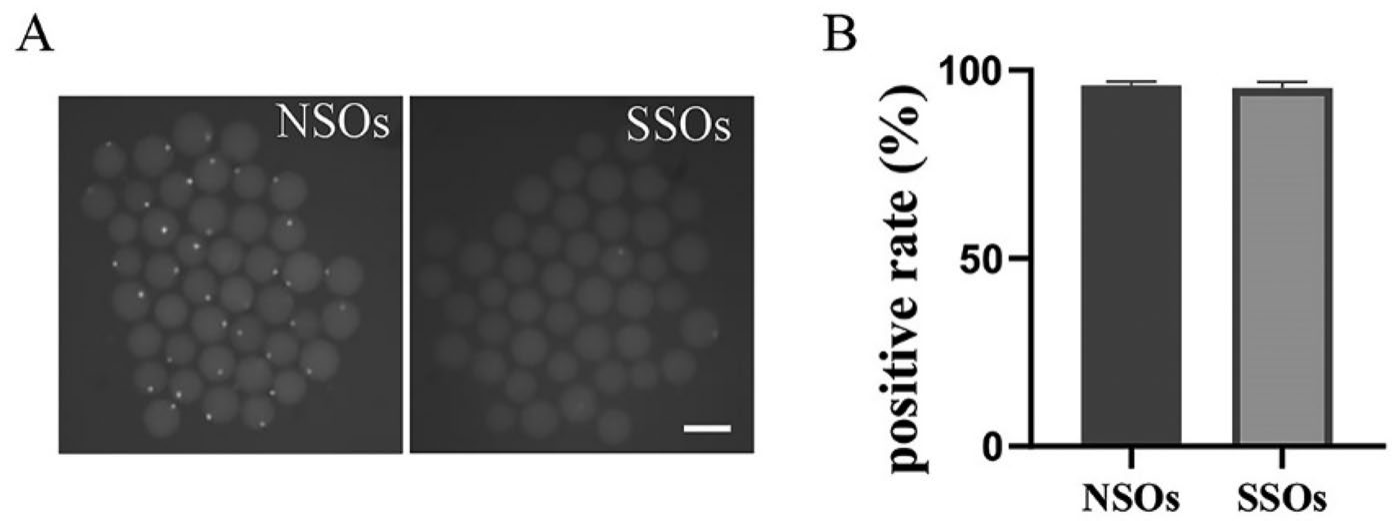
Quality control of NSOs and SSOs. (A) Nucleus were checked by hoechst 33342 staining. Bar = 80 μm. (B) The positive rates of karyoplasts in NSOs and cytoplasts in SSOs.

After the oriented bisection as shown in Fig. 2A, karyoplasts and cytoplasts were collected for NSO and SSO groups, respectively. As shown in Fig. 2B, pig matured oocytes could be bisected into two parts of NSO and SSO efficiently, that the positive rate of karyoplasts in NSOs was 95.91 ± 1.09% and the positive rate of cytoplasts in SSOs was 95.30 ± 1.63%.

### Transcriptome data in NSOs and SSOs

**Figure 3.**
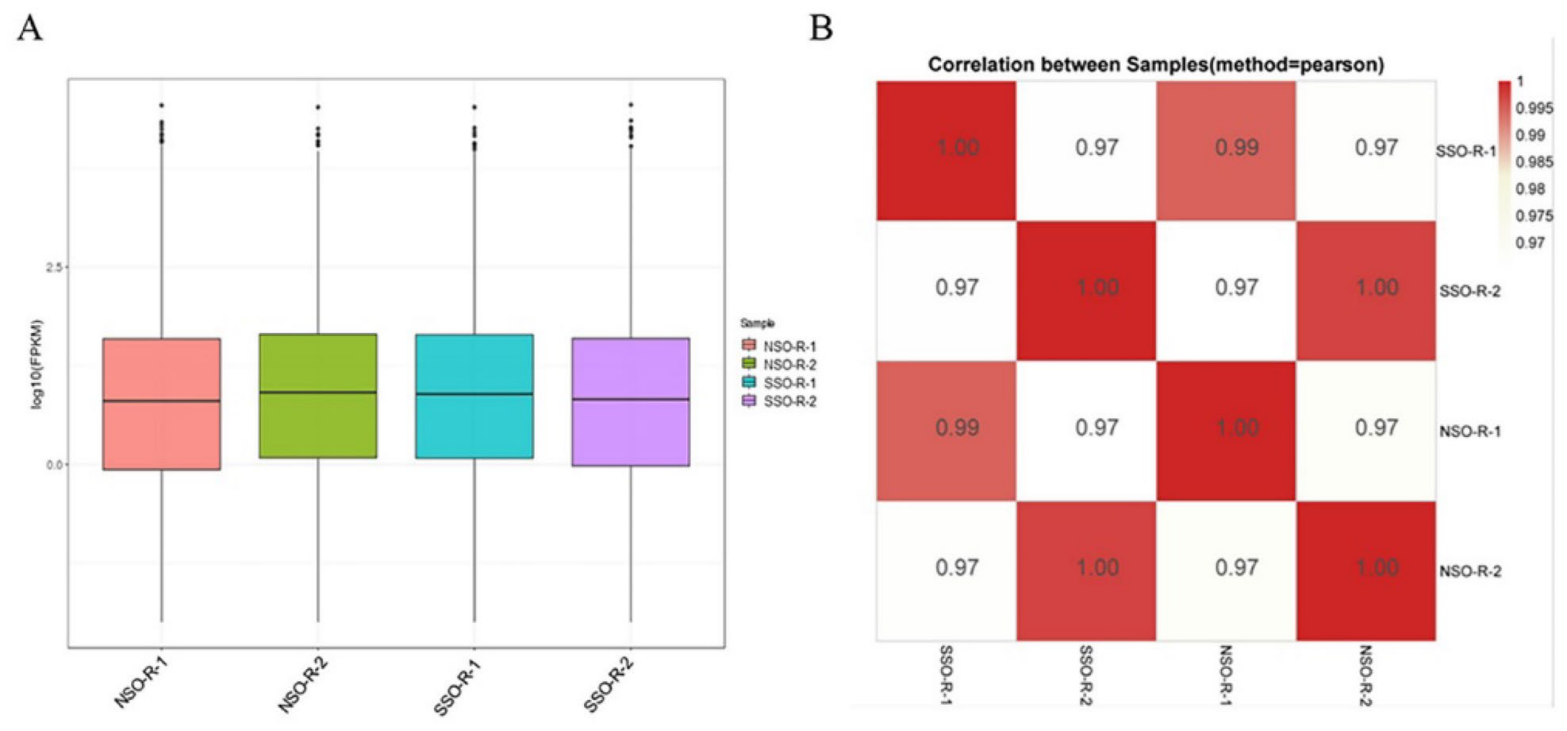
Transcriptome analysis. (A) Gene expression level statistics. (B) Pearson’s correlation coefficient analysis of quantitative results.

To fully verify the mRNA in NSOs and SSOs, RNA-Seq was carried out in NSO-R (NSO-R-1 and NSO-R-2) and SSO-R (SSO-R-1 and SSO-R-2) groups. The statistics of the raw data and the clean data were showed in Tables 1 and 2, respectively. Approximately 85% of the clean-reads were mapped onto pig reference genome (version: Sus scrofa11.1) that the total mapped reads ranged from 37,797,204 to 67,128,982 with a rate of 84.10%–86.90%, and the ratio of mapped pair reads was 75.20%–77.80% (Table 3). As shown in Fig. 3A, no significant difference was observed of all mRNAs in all groups, and pearson correlation coefficients between NSO-R groups (NSO-R-1 and NSO-R-2) vs. SSO-R groups (SSO-R-1 and SSO-R-2) were 0.99 and 1 (Fig. 3B). According to |log2Ratio| ≥ 1 and FDR-value ≤ 0.05, no DEG was detected between NSO-R groups and SSO-R groups.

**Table 1.** Statistics of the raw data.

Sample	Reads Number	Base Number(bp)	Length (bp)	Q20 (%)	Q30 (%)
NSO-R-1	72,310,340	10,846,551,000	150	95.07	88.39
NSO-R-2	40,475,822	6,071,373,300	150	95.96	90.48
SSO-R-1	64,373,936	9,656,090,400	150	95.19	89.15
SSO-R-2	40,397,996	6,059,699,400	150	95.87	90.28

**Table 2.** Statistics of the clean data.

Sample	Reads Number	Base Number (bp)	Q20 (%)	Q30 (%)
NSO-R-1	67,128,982	10,069,347,300	96.56	90.26
NSO-R-2	37,862,414	5,679,362,100	97.29	92.14
SSO-R-1	59,029,316	8,854,397,400	96.92	91.33
SSO-R-2	37,797,204	5,669,580,600	97.2	91.93

**Table 3.** Mapping results with pig genome.

Sample ID	Total Reads	Total Mapped Reads	Mapped Pair Reads	Mapped Left Reads	Mapped Right Reads
NSO-R-1	67,128,982	84.10%	75.20%	28,213,452 (84.1%)	28,243,244 (84.1%)
NSO-R-2	37,862,414	86.90%	77.80%	16,343,952 (86.3%)	16,550,447 (87.4%)
SSO-R-1	59,029,316	84.20%	75.30%	25,153,581 (85.2%)	24,539,987 (83.1%)
SSO-R-2	37,797,204	86.60%	77.40%	16,026,896 (84.8%)	16,687,776 (88.3%)

### Proteomics data in NSOs and SSOs

**Figure 4.**
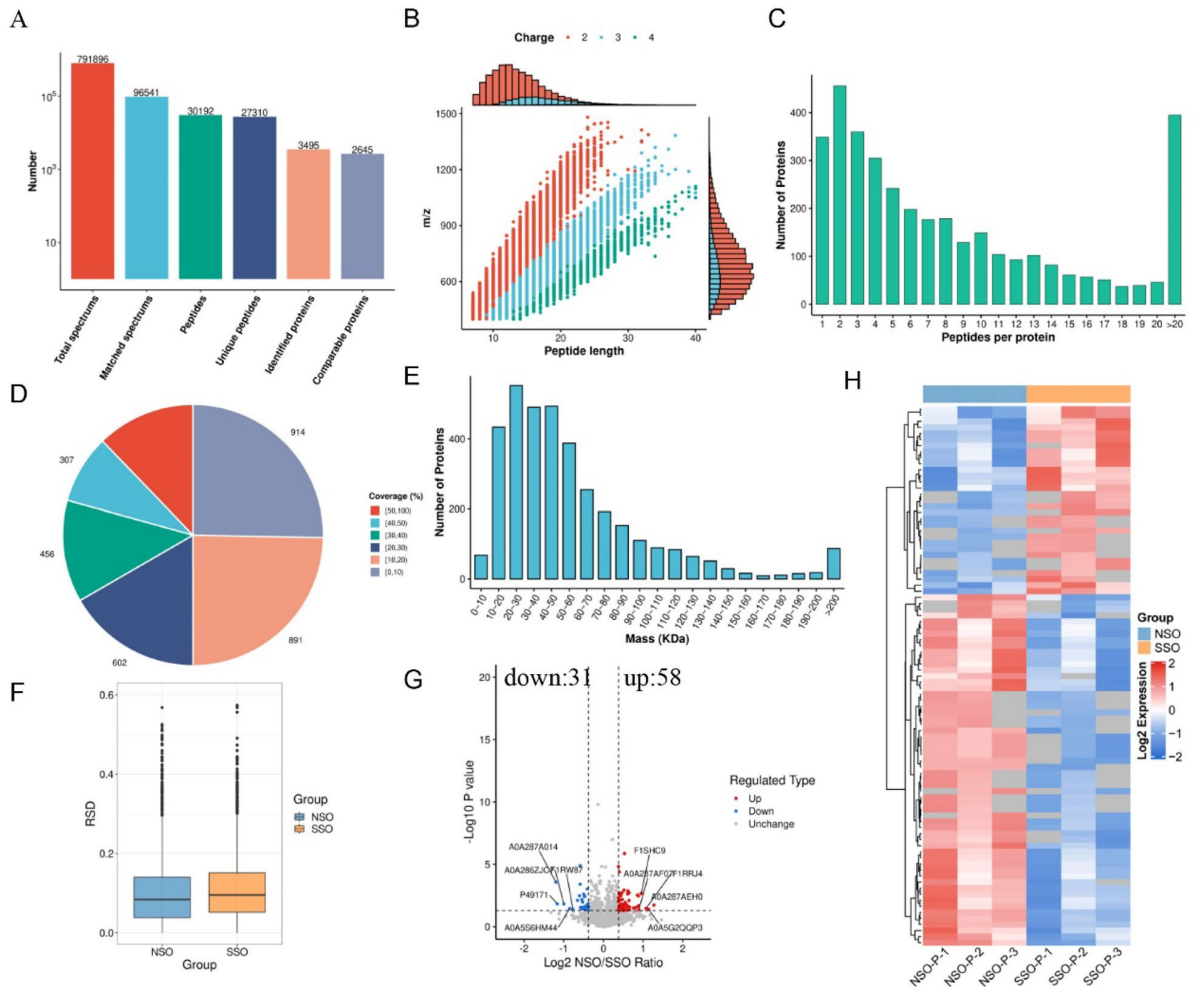
Quantitative proteome analysis and identification of DEPs. (A) Summary of tandem mass spectrometry database searches analysis. (B) Length distribution of all identified peptides. (C) Number distribution of peptides per protein. (D) Distribution of protein coverage. (E) Distribution of proteins molecular mass. (F) Relative standard deviation (RSD) distribution of repeated samples. (G) Volcano plot of DEPs. (H) Heat map showing the hierarchical clustering of DEPs. The expression of DEPs in different samples is shown with red (high expression) and blue (low expression).

As shown in Fig. 4A, 3495 proteins were identified from groups of NSO-P (NSO-P-1, NSO-P-2 and NSO-P-3) and SSO-P (SSO-P-1, SSO-P-2 and SSO-P-3), but 2645 proteins from which were quantified. Most peptides of all identified proteins from NSO-P groups and SSO-P groups were 7–20 amino acids, in line with the general law based on enzymatic lysis and mass spectrometry fragmentation (Fig. 4B). Based on two or more peptides, most proteins were identified as shown in Fig. 4C, with less than 30% coverage (Fig. 4D), and molecular weight of which was in the range of 10–200 kDa (Fig. 4E). The relative standard deviation (RSD) of protein quantitative values between replicates in the same groups of NSO-P (NSO-P-1 vs. NSO-P-2 vs. NSO-P-3) or SSO-P (SSO-P-1 vs. SSO-P-2 vs. SSO-P-3) was less than 0.2 (Fig. 4F). A total of 89 proteins with *p*-value < 0.05 and fold change (FC) >1.30 or < 0.76 were identified as DEPs between NSO-P and SSO-P groups, of which 58 proteins were up-regulated DEPs, and 31 proteins were down-regulated DEPs in the NSO-P groups (Fig. 4G). The hierarchical clustering of the DEPs is partitioned into two distinct clusters (Fig. 4H).

### Functional categories of DEPs

**Figure 5.**
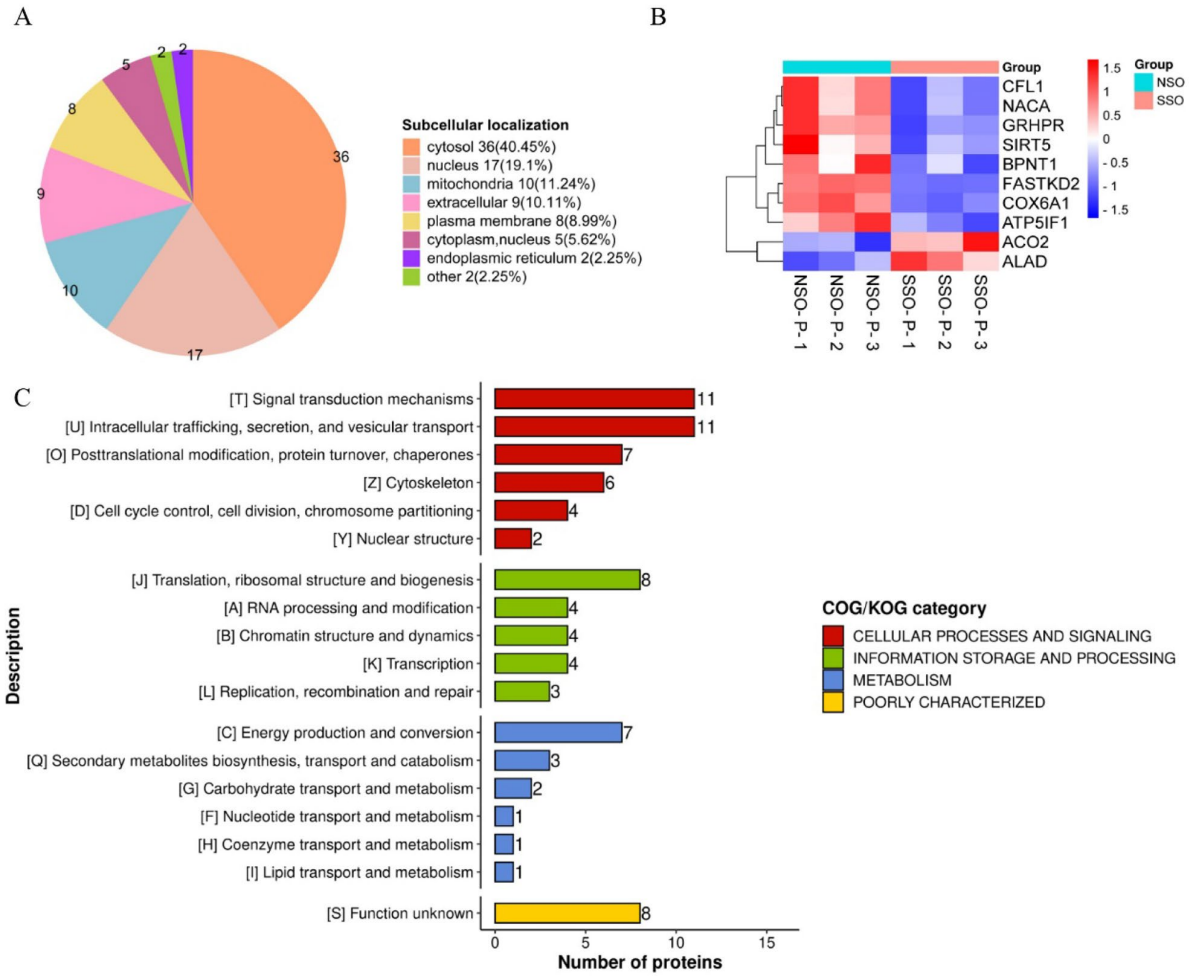
Subcellular localization and COG/KOG functional classification of DEPs. (A) Subcellular localization classification of DEPs. (B) Heat map analysis of DEPs localized to mitochondria. (C) COG/KOG functional classification of DEPs.

Subcellular structural annotation of DEPs was performed as shown in Fig. 5A, it is worth to note that 11.24% of DEPs located in mitochondria, including ATP5IF, GRHPR, NACA, FASTKD2, SIRT5, COX6A1, CFL1 and BPNT1 (Fig. 5B). The functional classification of DEPs being analyzed by COG/KOG (Fig. 5C) showed that the largest category with 11 DEPs is ‘signal transduction mechanisms’ and ‘intracellular trafficking, secretion, and vesicular transport’, followed by ‘translation, ribosomal structure and biogenesis’ with 8 DEPs, ‘energy production and conversion’ with 7 DEPs, ‘posttranslational modification, protein turnover, chaperones’ with 7 DEPs, and ‘cytoskeleton’ with 6 DEPs.

### GO and KEGG enrichment analysis of DEPs

**Figure 6.**
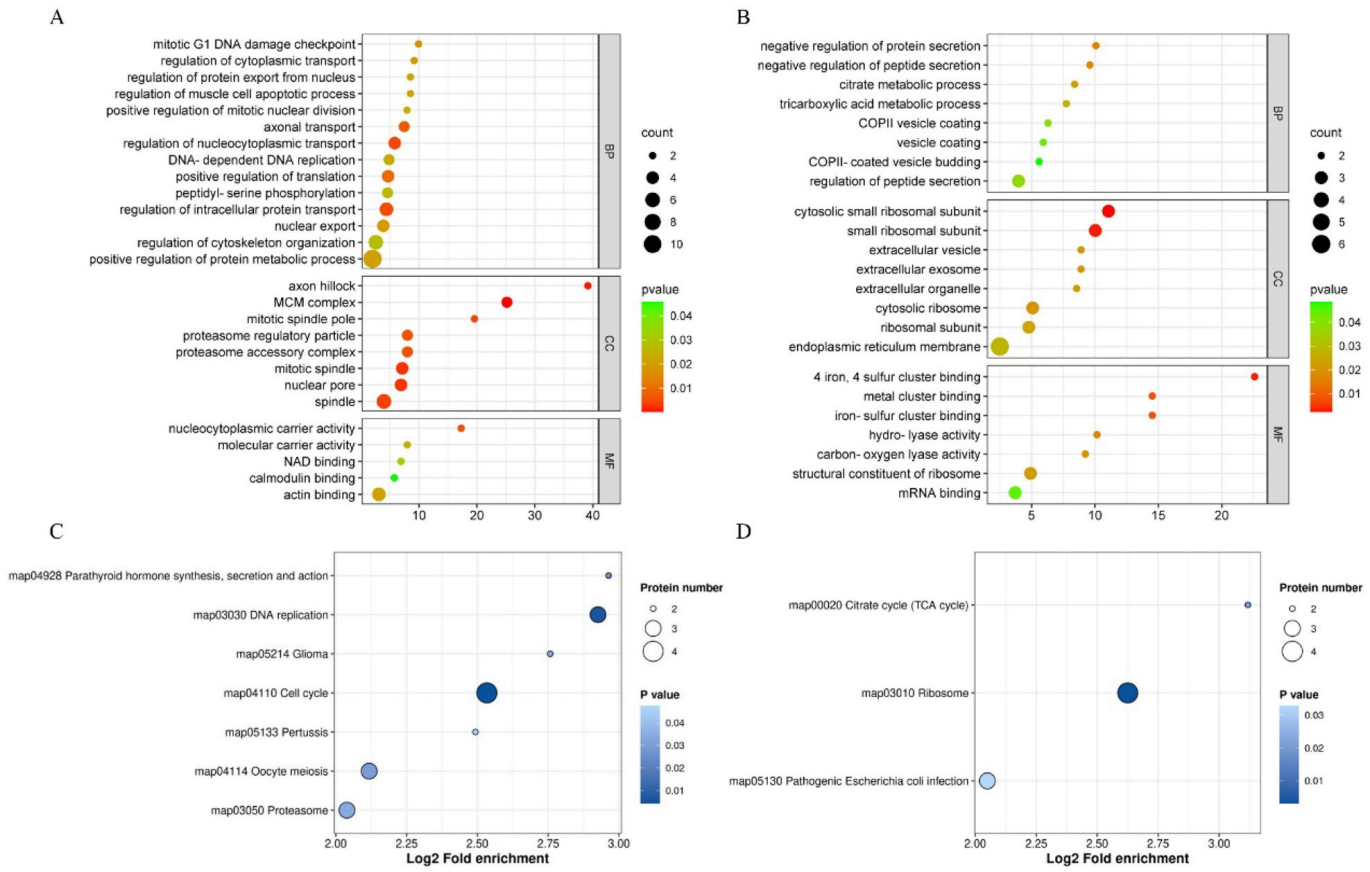
GO and KEGG analysis of DEPs. (A) GO enrichment analysis of up-regulated DEPs. (B) GO enrichment analysis of down-regulated DEPs. (C) KEGG pathway enrichment analysis of up-regulated DEPs. (D) KEGG pathway enrichment analysis of down-regulated DEPs.

Functional enrichment analyses including GO and KEGG pathway were carried out. The most up-regulated DEPs in NSOs were GO for ‘spindle’, ‘regulation of nucleocytoplasmic transport’, ‘positive regulation of translation’, ‘DNA-dependent DNA replication’, ‘regulation of cytoskeleton organization’ and ‘mitotic G1 DNA damage checkpoint’ as shown in Fig. 6A, and the down-regulated DEPs were GO for ‘cytosolic small ribosomal subunit’, ‘negative regulation of protein secretion’ and ‘mRNA binding’ as shown in Fig. 6B. In addition, the KEGG pathway revealed that DEPs of up-regulated in NSO-P groups were associated with cell cycle, including ‘cell cycle’, ‘DNA replication’ and ‘oocyte meiosis’, but the down-regulated DEPs in NSO-P groups were in ‘ribosome’ and ‘citrate cycle (TCA cycle)’ pathways (Fig. 6C and D).

### The identification of DEPs

**Figure 7.**
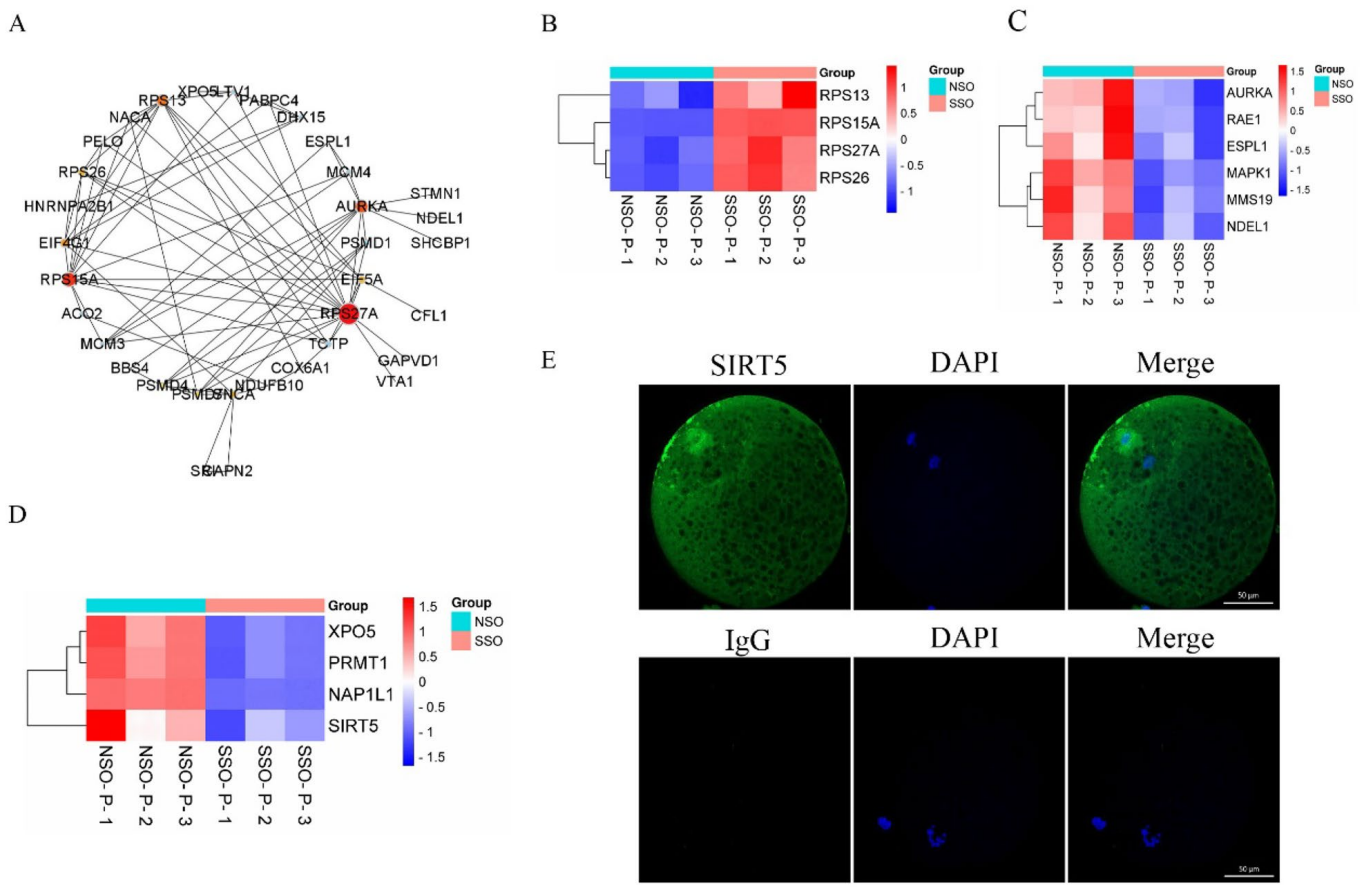
PPI network and verification of DEPs. (A) PPI network of DEPs. The circle size represents the degree of protein interaction. (B) Heat map analysis of DEPs involved in ribosome signaling pathway. (C) Heat map analysis of DEPs involved in spindle function. (D) Heat map analysis of DEPs of SIRT5, XPO5, PRMT1 and NAP1L1. (E) IF result of SIRT5 protein. Bar = 50 μm.

The PPI network analysis was used to elucidate the functional interactions of DEPs as shown in Fig. 7A. A down-regulated protein RPS27A had the highest connectivity, and proteins that interacted with it including RPS15A, RPS13 and RPS26 were involved in the ribosome signaling pathway (Fig. 7B). And another six proteins involved in spindle function were identified (Fig. 7C). As a representative up-regulated DEP, Aurora Kinase A (AURKA) was GO in 130 items such as ‘meiotic cell cycle process’, ‘nuclear division’, and ‘regulation of cellular component organization’ (Supplementary Table 1). In addition, four important maternal proteins (SIRT5, XPO5, PRMT1 and NAP1L1) have also been identified (Fig. 7D). The up-regulation DEP of SIRT5 widely involved in the ‘regulation of gene expression, epigenetic’ (Supplementary Table 1), were further immunofluorescence confirmed the accumulation of SIRT5 around the nucleus (Fig. 7E).

## Discussions

In the era of experimental manipulation and the increasing popularity of in vitro fertilization, it is crucial to understand the asymmetry/polarity of mature mammalian oocytes.^30^ Localization of maternal factors has been widely observed in lower animals. However, it is not clear whether the polarity of maternal factors existed in the cytoplasm of mammalian MII oocytes. Although no difference of mRNA could be detected in pig matured oocytes in the present study, an uneven distribution of proteins between NSOs and SSOs was detected. That these DEPs highly expressed in NSOs mainly involved in spindle function, DNA replication, and cell cycle correlation while ribosome-related proteins and RNA-binding proteins were significantly enriched in SSOs. Therefore, the DEPs highly expressed in NSOs were supposed to play key roles in the formation and function of the spindle and the separation of chromosome during meiosis. DEPs significantly enriched in SSOs might directly affect the pre-cleavage patterning of these intracellular materials.

### Asymmetry distribution of proteins rather than mRNA are present in MII porcine oocytes

After a period of transcriptional activity during growth, the transcriptional activity of mammalian oocyte nuclei (germinal vesicle, GV) is inhibited.^31^ In the absence of transcription, meiosis and early embryonic development in mammals are heavily dependent on mRNAs and proteins accumulation by the maternal line.^32^ Local mRNA plays an important role in the establishment, function, and regulation of cell division mechanisms.^33^^,^^34^ Commonly, samples of the spindle chromosome complex were mainly isolated by microsurgery for microarray analysis. However, with the modification of steps in HMC,^16^ it is easier to collect enough semi-oocytes by the oriented bisection, which would provide an efficient way for the analysis on the distribution of maternal factors in oocytes. A polarities localization pattern of mRNA was noticed in *drosophila*,^34^ but very few studies are available on mammalian oocytes. It is noticed that a difference expression of mRNA existed in mouse oocytes,^8^^,^^9^ but no DEGs were detected in NSOs and SSOs of pig oocytes. It might because that the translation of mRNA is spatially limited to microtubules but not enriched on the spindle.^35^ And local library RNA pools of specific transcripts supporting various spindle functions remain in the area of spindle formation, after the migration of the spindle from the oocyte center to the cortex.^36^ In fact, the spindle in meiosis is not only the central structure involved in the polarization of the oocyte but also becomes an active vector bringing the cortical proteins necessary for asymmetric division (such as CDK1, ERK1/2, and mPARD6a and b) to the right place.^30^ Thus, the final polarity of the mature oocyte involves selective translation and carrier capacity in spindle migration.

### Spatial distribution of proteins of MII oocytes might affect the development of pig embryos

During meiosis of mammalian oocytes, overall protein synthesis is gradually reduced.^36^ Although various regulatory mechanisms such as reconstruction of spindle and cytoskeleton, epigenetic modification and energy metabolism have been revealed by the recent proteomic profiles of porcine oocytes,^37^ these proteins were detected continuously accumulated with maturation to maintain the normal development of oocytes. However, the spatial distribution of maternal factors in the matured oocytes was very limited. The present study was firstly proteomic analysis for the oriented semi-oocytes, and key proteins of AURKA, RAE1, ESPL1, MAPK1, MMS19 and NDEL1 were identified as DEPs. As a protein with the highest connectivity, AURKA is a member of the serine/threonine protein kinase family^38^ involved in chromosome separation and meiosis, and needed for spindle construction at the beginning of meiosis recovery.^39^^,^^40^ Combined with GO function analysis, the present results might indicate that the presence of local translation within oocytes that requires special proteins to support spindle formation, maintenance, and function, and further these proteins involved in spindle migration to the oocyte cortex and spindle rotation after fertilization.^41^^,^^42^

Another three proteins of RPS27A, RPS13 and RPS26 detected as DEPs were enriched in the ribosome signaling pathway. Actually, the pathway of ribosome signaling regulates cell cycle processes, cell growth, proliferation and survival, and plays an important role in maintaining the function of oocytes.^43^ The mRNA level of RPS27A in buffalo oocytes is significantly higher with maturation, indicating that RPS27A may play an important role in the maturation of oocytes.^44^ RPS13 and RPS26 participate in mRNA binding and are related to selective translation.^45^^,^^46^ Hence, the differential distribution of proteins participated in the pathway of ribosome signaling between NSO and SSO may account for the heterogeneity of blastomere after the first cleavage due to uneven distribution,^47^^,^^48^ and further affect the following development of embryos.

### Spatial distribution of proteins in MII oocytes might be the maternal factors important for reprogramming

It has been reported that the low developmental ability of SCNT embryos is associated with the deficient spindle composition due to the deletion of four proteins Clathrin heavy chain (CLTC), Aurora B kinase, dynactin 4 and casein kinase 1α.^49^ The above six functional spindle DEPs of AURKA, RAE1, ESPL1, MAPK1, MMS19 and NDEL1 identified highly expressed in NSOs might be removed when enucleation of SCNT, that resulted in the spindle defects in cloned embryos, and further affect the development of cloned embryos. In addition, four maternal proteins (SIRT5, XPO5, PRMT1 and NAP1L1) were found up-regulated in NSOs, and all these four proteins play important role in reprogramming. It is known that SIRT5 has mitochondrial localization and regulates different pathways, including glucose oxidation, ketone body formation, fatty acid degradation, ammonia treatment, and REDOX homeostasis,^50^ and SIRT5 appears to be a key determinant of stem cell differentiation and reprogramming.^51^ PRMT1-dependent H4R3 methylation (R3me2a) allows the recruitment of the Tudor domain-containing protein, TDRD3, which in turn associates with topoisomerase IIIβ (Top IIIβ) to reduce R-loop formation and RNA polymerase II (RNA pol II) to promote transcriptional activity.^52^ NAP1L1 was reported could enhance chromatin remodeling^53^ and mediate nucleosome formation or disassembly by associating with various histone.^54^^,^^55^ During reprogramming, a large number of nucleosomes will firstly disassemble and then reassemble,^56^ and then histone variants such as H3.3 and H2AX can be incorporated into nucleosomes to promote somatic nuclear reprogramming.^18^ Therefore, the present study firstly indicated that the removal of partial cytoplasm of matured oocytes might result in the deletion of the main material factors crucial for the reprogramming of somatic cell during SCNT, which further affect the development of cloned embryos, and it is crucial to know the DEPs of the oriented semi-oocyte.

In conclusion, the present confirmed a spatial asymmetry of maternal factors at the protein level in pig oocytes using the multi-omics analysis on the semi-oocytes achieved by the oriented bisection of oocytes. This spatial asymmetry of maternal factors in pig oocytes could provide a reference for improving reprogramming and cloning efficiency and the origin of 2-cell heterogeneity.

## Supplementary Material

Supplementary Table 1.xlsx
